# Comparison of Calculations of the Financial Impact of Fellowship Training by Data Source

**DOI:** 10.1001/jamanetworkopen.2023.26639

**Published:** 2023-07-28

**Authors:** Gary L. Freed, Kyle Wickham

**Affiliations:** 1 Department of Pediatrics Division of General Pediatrics, Susan B. Meister Child Health Evaluation and Research Center, University of Michigan Health Systems, Ann Arbor; 2 University of Michigan Medical School, Ann Arbor

## Abstract

**Background:**

Previous comparisons of potential lifetime earnings between general pediatricians and pediatric subspecialties have demonstrated that many subspecialties have lower potential lifetime earnings than general pediatrics. However, those studies selectively used specific data sources for different portions of analyses.

**Objective:**

To assess the presence and magnitude of differences in earning forecasts using different authoritative data sources.

**Methods:**

This quality improvement study analyzed compensation data for 14 pediatric subspecialties and general pediatrics from the Association of Administrators in Academic Pediatrics (AAAP), Association of American Medical Colleges (AAMC), and Medical Group Management Association (MGMA) for the 2021 to 2022 academic year.

**Main Outcomes and Measures:**

The potential lifetime compensation was calculated using the net present value (NPV), which accounts both for compensation throughout training (residency and fellowship) and compensation after graduation. The potential lifetime compensation for the subspecialties and general pediatrics was compared separately for each data source.

**Results:**

This study included data from 3 sources about 14 subspecialties and general pediatrics. Depending on the data source, the magnitude of the difference in lifetime earnings between subspecialties and general pediatrics varied greatly. For all sources, there was a greater difference in lifetime earnings between higher- and lower-paid subspecialties compared with each other relative to general pediatrics. For the AAAP, the subspecialty with the greatest lifetime NPV is neonatal medicine, and the subspecialty with the least lifetime NPV is endocrinology, with a difference of $2 787 539. For the AAMC, cardiology has the greatest lifetime NPV and endocrinology the least, with a difference of $3 557 492. For the MGMA, neonatal medicine has the greatest lifetime NPV and adolescent medicine the least, with a difference of $4 210 477. Additionally, there is a notable difference in lifetime earnings in private vs academic practice.

**Conclusions and Relevance:**

These findings suggest that the difference in lifetime compensation between many pediatric subspecialties and general pediatrics is not as large as previously reported. Also, greater differences exist when comparing private practice vs academic medicine and between higher- and lower-paid subspecialties.

## Key Points

**Question:**

Do different national data sources provide diverse results for lifetime earnings of the subspecialties of pediatrics?

**Findings:**

In this quality improvement study using data from 3 sources, the magnitude of the difference in lifetime earnings between subspecialties and general pediatrics varied greatly. For all sources, there was a greater difference in lifetime earnings between higher- and lower-paid subspecialties compared with each other relative to general pediatrics.

**Meaning:**

These findings suggest that the difference in lifetime compensation between pediatric subspecialties and general pediatrics is not as large as previously reported.

## Teaser

This quality improvement study analyzes compensation data of 14 pediatric subspecialties and general pediatrics from 3 data sources to determine if different data sources provide varied results.

## Introduction

The decision by pediatricians to pursue fellowship training, and if so, which subspecialty to choose, has been a focus of interest among leaders in the field.^1,2^ Perceptions of shortages in specific subspecialties have created an urgency in determining the factors informing this decision and resulted in a plethora of potential policy options to designed to impact the pediatric workforce, mostly in the financial arena.^3,4,5,6,7^ However, many studies have demonstrated that residents do not report that potential compensation is a primary factor in their decision to pursue specific fellowship training^8,9,10,11^; yet, the accuracy of such disclosures is unknown. Although previous research has demonstrated that the decision regarding whether or not to pursue fellowship training is made during their internship or even earlier for most pediatricians,^12^ there has also been considerable interest in the impact that experiences and knowledge of the subspecialties during residency can play in this process. However, the overall number of trainees pursuing fellowship training has increased every year for at least the past 30 years,^13^ the proportion pursuing specific subspecialties is not equally distributed.^14^

Recently, several studies have highlighted differences in the potential compensation of those who choose to undertake fellowship training compared with general pediatricians.^8,15^ Financial considerations are known to play a role in a host of decisions made throughout life, and such studies hypothesize that this compensation differential may impact the decision to pursue subspecialty training in general or in a specific subspecialty.^1,6,15^ If financial considerations are an important part of the decision process to pursue fellowship training, then it is essential to ensure a clear understanding of the presence and magnitude of these financial implications.

There are 3 primary data sources for pediatrician compensation based on employer-provided information rather than surveys of pediatricians themselves. These are the American Association of Administrators in Pediatrics (AAAP), the American Association of Medical Colleges (AAMC), and the Medical Group Practice Association (MGMA). Previous studies have combined some of these sources to illustrate differences in potential compensation among generalists and subspecialists.^8,15^ However, using data from 1 source for some pediatricians and a different data source for others may impact results markedly, especially when significant differences exist between the sources. We hypothesized that conducting the same analyses using different data sources would yield different results.

To test this hypothesis, we have repeated previous studies^8,15^ using these 3 data sources independently. We believe this approach may provide a clearer picture of the range of differences in potential compensation for general pediatrics and the subspecialties and shed light on the implications for using seemingly valid but different data sources for the same analyses.

Additionally, missing from previous analyses has been an assessment of the magnitude of differences in potential lifetime compensation among the subspecialties themselves. If financial return is believed to be a motivating force in the decision process of whether to pursue fellowship training, it follows that the choice of a specific subspecialty may also be affected in a similar fashion. If so, such information is an important part of workforce analysis and planning.

## Methods

This quality improvement study followed the Standards for Quality Improvement Reporting Excellence (SQUIRE) reporting guideline. Approval and informed consent were waived by the University of Michigan Medical School institutional review board because there were no human participants involved in this study.

Our methods were based on previous work analyzing the differences in lifetime earnings among pediatric subspecialists.^15^ Using data singly from each of the 3 income sources of practicing pediatricians, the mean income of general pediatricians and each pediatric subspecialties were used to create a model that estimates the net income during a working career. In addition to general pediatrics, the subspecialties included were adolescent medicine, cardiology, child development, critical care, emergency medicine, endocrinology, gastroenterology, hematology and oncology, hospital medicine, infectious disease, neonatal medicine, nephrology, neurology, pulmonology, and rheumatology.

### Sources of Compensation Data

Resident and fellowship stipend and physician annual salary data were obtained for the academic year 2021 to 2022. Data from the AAMC Survey of Resident/Fellow Stipends and Benefits report were used for resident and fellowship stipends.^16^

Analyses for annual salary after residency for general pediatrics or after residency and fellowship for subspecialties were conducted using data from the AAAP, the AAMC, and the MGMA. The AAAP provides data from more than 100 academic pediatric departments and reports the total annual compensation, including bonuses and before taxes, retirement, and fringe benefits, and reflects salaries from the 2021 to 2022 academic year. For pediatric cardiology, diagnostic cardiology data were used. The AAMC provides data on faculty associated with medical schools, thus limiting the sample to those practicing in academic settings. The AAMC data reports annual total compensation, including bonuses and before taxes, retirement, and fringe benefits. Data from the AAMC reflected the fiscal year 2021 salaries. The MGMA provides income data that represents pediatricians in multiple practice settings, including private practice and academic medicine. The MGMA data set reports the annual total compensation, including bonuses and before taxes, retirement, and fringe benefits. Data from the 2021 MGMA report were used. Analyses for child development were conducted using data from AAAP and MGMA, but the AAMC did not have income data on this subspecialty, so it was excluded from AAMC analyses.

For the analyses of AAAP and AAMC data, we used assumptions made in previous published studies that subspecialists worked for 7 years as an assistant professor, 7 years as an associate professor, and the rest of their career as a full professor after fellowship.^15^ For of the MGMA data, as in previous studies, the mean income for general pediatrics and each subspecialty was used.

### Statistical Analysis

To estimate the potential lifetime earnings as in the Catenaccio et al,^17^ we used the net present value (NPV), which is a financial technique used to determine the value of an investment, taking into account future and past cash flows. By doing this, we can estimate the potential lifetime earning of those pursuing a fellowship for a career as a pediatric subspecialist and compare that with the potential lifetime earning of a graduating resident not pursuing fellowship and practicing as a general pediatrician after residency. The formula for NPV is:



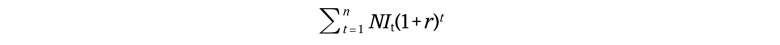



where *NI* is the annual net income and *r* is the discount rate, which was set at 1% based on the US discount rate as of May 2022.^18^ This formula takes the sum of the annual net income over time (*t* = 1) to n years. We assumed that no time was taken off between high school, undergraduate college, medical school, residency, fellowship, and full-time employment. For subspecialists, we assumed a standard fellowship length of 3 years for all subspecialties. Of note, hospital medicine fellowships are typically 2 years long, and pediatric neurology is often a combined 5-year residency program; however, we assumed a 3-year residency length and 3-year fellowship length for these subspecialties as in previous reports.^15^ After completion of residency or fellowship, we assumed that graduates worked as full-time employees with an equal number of hours worked per year until a retirement age of 65. Microsoft Excel version 2304 (Microsoft) was used for statistical analysis.

## Results

**Figure 1.**
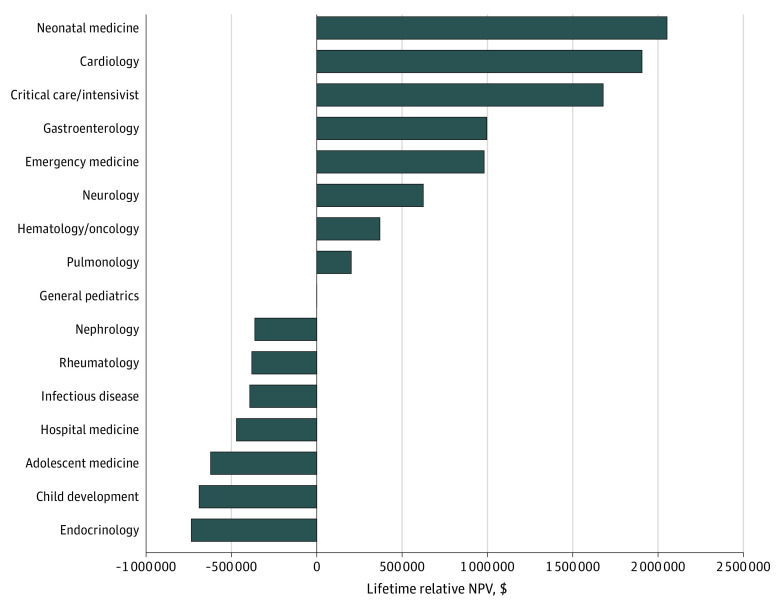
Lifetime Relative Net Present Values (NPVs) by Subspecialty According to the Association of Administrators in Academic Pediatrics

This study includes data from 14 pediatric subspecialties and general pediatrics from the AAAP, AAMC, and MGMA. Figure 1 illustrates the lifetime relative NPV earnings of 14 pediatric subspecialties and general pediatrics using data from the AAAP, representing the salaries of academic pediatricians.^19^ In this analysis, the lifetime relative NPV for general pediatrics was set to $0, and the pediatric subspecialist NPVs were compared with general pediatrics. The subspecialties with positive potential lifetime earnings compared with general pediatrics were neonatal medicine, cardiology, critical care or intensivist, gastroenterology, emergency medicine, neurology, hematology, and pulmonology. The subspecialty with the greatest positive lifetime relative NPV was neonatal medicine, which has a $2 052 776 positive potential lifetime earnings compared with general pediatrics. The specialties with negative potential lifetime earnings relative to general pediatrics were nephrology, rheumatology, infectious diseases, hospital medicine, adolescent medicine, child development, and endocrinology, with the largest negative NPV seen in endocrinology at $734 764. In other words, the potential lifetime earning of an academic endocrinologist was $734 764 less than an academic general pediatrician.

**Figure 2.**
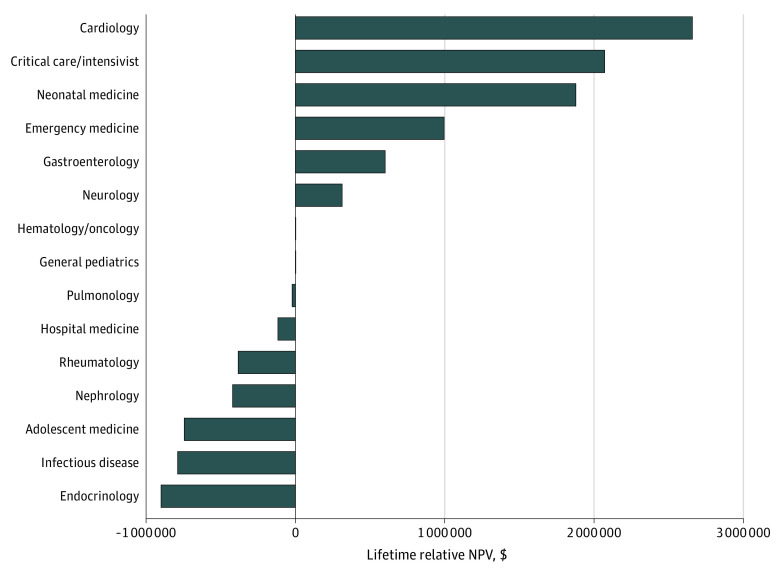
Lifetime Relative Net Present Values (NPVs) by Subspecialty According to the Association of American Medical Colleges

Similar to Figure 1, Figure 2 illustrates the lifetime relative NPV of 14 pediatric subspecialties to general pediatrics using data from the AAMC, which also represents the salaries of academic pediatricians. The subspecialties with positive potential lifetime earnings compared with general pediatrics were cardiology, critical care or intensivist, neonatal medicine, emergency medicine, gastroenterology, neurology, and hematology or oncology. In contrast to AAAP data, using AAMC data, the subspecialty with the greatest lifetime relative NPV was cardiology, which had a $2 657 620 positive potential lifetime earnings compared with general pediatrics. The specialties with negative potential lifetime earnings compared with academic general pediatrics were pulmonology, hospital medicine, rheumatology, nephrology, adolescent medicine, infectious disease, and endocrinology, with the largest negative NPV in endocrinology at $899 871.

**Figure 3.**
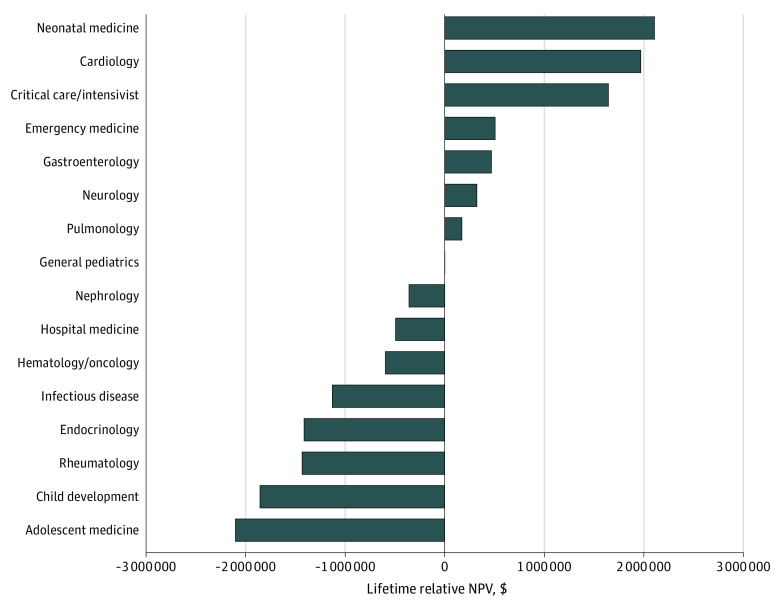
Lifetime Relative Net Present Values (NPVs) by Subspecialty According to the Medical Group Practice Association

Figure 3 uses data from the MGMA, which provides blended salaries from academic and private practice settings. In this analysis, the subspecialties with positive potential lifetime earnings compared with general pediatrics were neonatal medicine, cardiology, critical care or intensivist, emergency medicine, gastroenterology, neurology, and pulmonology. The subspecialty with the greatest lifetime relative NPV was neonatology which had a $2 106 711 positive potential lifetime earnings vs general pediatrics. The subspecialties with less potential lifetime earnings compared with general pediatrics were nephrology, hospital medicine, hematology or oncology, infectious diseases, endocrinology, rheumatology, child development, and adolescent medicine. In contrast to both the AAAP and the AAMC, the subspecialty with the largest negative NPV was adolescent medicine at $2 103 764.

**Figure 4.**
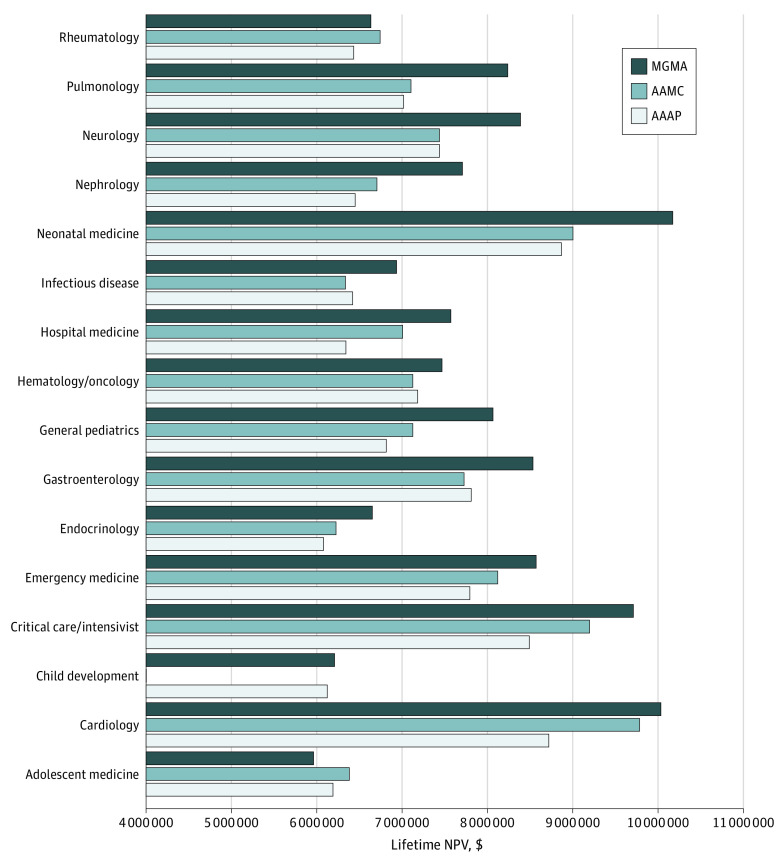
Comparison of Lifetime Net Present Values (NPVs) by Data Source AAAP indicates Association of Administrators in Academic Pediatrics; AAMC, Association of American Medical Colleges; MGMA, Medical Group Practice Association.

Figure 4 compares the lifetime NPV for general pediatrics and each subspecialty using the 3 data sources. For all subspecialties except for adolescent medicine and rheumatology, the lifetime NPV was highest using MGMA data. Several subspecialties hadw marked differences in lifetime NPV of more than $1 000 000 between the different data sources. These were cardiology, critical care and intensivist, hospital medicine, neonatal medicine, nephrology, pulmonology, and general pediatrics. The subspecialties with the smallest difference in lifetime NPV (less than $500 000) between the different data sources were adolescent medicine, child development, hematology or oncology, and rheumatology. Figure 4 also shows that comparisons between general pediatrics and subspecialties that use different data sources for each can result in higher or lower relative lifetime NPV assessments.

**Figure 5.**
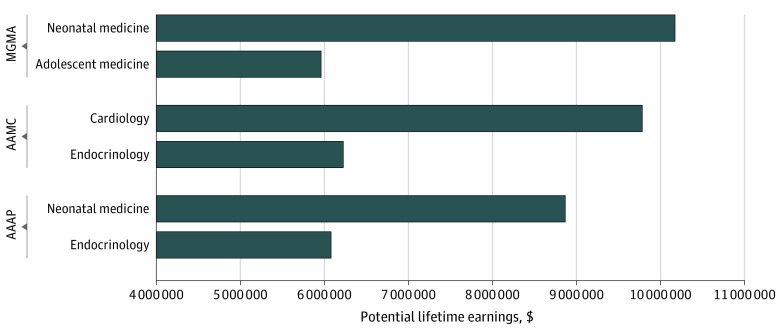
Subspecialties With the Highest and Lowest Potential Lifetime Earnings for Each Data Source AAAP indicates Association of Administrators in Academic Pediatrics; AAMC, Association of American Medical Colleges; MGMA, Medical Group Practice Association.

Figure 5 displays the subspecialties with the greatest and least lifetime NPV for each data source. For the AAAP, the subspecialty with the greatest lifetime NPV was neonatal medicine, and the subspecialty with the least lifetime NPV is endocrinology, with a difference of $2 787 539. For the AAMC, the subspecialty with the greatest lifetime NPV was cardiology, and the subspecialty with the least lifetime NPV was endocrinology, with a difference of $3 557 492. For the MGMA, the subspecialty with the greatest lifetime NPV was neonatal medicine, and the subspecialty with the least lifetime NPV was adolescent medicine, with a difference of $4 210 477.

## Discussion

One important finding from this study is that the differences in lifetime compensation between many of the subspecialties, when compared with each other, are markedly greater than the differences between many subspecialties and general pediatrics. Another important finding is the notable variation in the differences in lifetime compensation between general pediatrics and the various subspecialties depending on the data source used.

Depending on the data source used, there were 6, 7, or 8 subspecialties with lifetime earnings less than those of general pediatricians. In contrast, when using 1 data source for general pediatrics and a different data source for the subspecialties, Catenaccio et al^17^ found 12 subspecialties to have lower lifetime earnings than general pediatrics. Specifically, that study illustrated AAMC data for all subspecialties, but MGMA data for general pediatrics. This choice appears to accentuate the differences in compensation in academic vs private practice and confound the differences between general pediatrics and subspecialties. In contrast, our analysis showed a much smaller difference in lifetime earnings between general pediatrics and the lowest compensated subspecialty for 2 of the 3 data sources examined.

Although general pediatricians are more likely to be in private practice than subspecialists, it is important to note that the proportion of those in private practice varies markedly by specific subspecialty. For example, more than half of all neonatologists are in private practice, and the rate is more than 25% for many other subspecialties.^20,21^ Thus, an aggregation bias (ecologic fallacy) can occur if data sources are selected to reflect that all of 1 group is engaged in academic employment and another in private practice, as seen in some previous studies.^8,15^ This may conflate the impact of differential remuneration of private practice with other factors, including specific specialty choice. Each data source provides different results when projecting the relationship of lifetime earnings among generalists and subspecialists, adding complexity to a seemingly simple concept. For example, even when only examining the AAAP and AAMC data for academic pediatricians, there is significant variability in lifetime compensation between the 2 sources for general pediatrics and almost all subspecialties.

Our results demonstrate that for all 3 data sources, some combination of subspecialties has a lower lifetime earning potential than general pediatrics. This raises the issue of whether, and potentially to what extent, this reality impacts the decision of trainees to pursue lower-paid subspecialties. Although financial advantage is a common motivation in society, it is not absolute. Whether these lower-paid subspecialties would have greater desirability if more generously compensated is, at this time, a theoretical question open to debate. Other factors, including a paucity of procedures and other potentially desirable types of work, also differentiate these lower-paid subspecialties from their higher paying counterparts. Before policy options are enacted and scarce resources allocated, a more definitive understanding of what would make matriculation into the lower compensated training programs more attractive is sorely needed. Such analyses should be conducted early in the training pathway to determine the factors informing the decision process of potential trainees, with follow-up conducted to assess potentially modifiable variables. Money may or may not be the prevailing factor. Existing studies of those in training, as opposed to those already in practice, suggest it is not.^12,22^

Another important issue raised in our analyses is the large disparities in lifetime compensation between many of the subspecialties; for example, there was more than a $4 million difference between neonatal medicine and adolescent medicine using the MGMA data. For many subspecialties, these differences dwarf the disparities in lifetime income with general pediatrics. Although differing in magnitude, these disparities exist across all 3 data sources, regardless of whether the subspecialists are in academic or private settings. At the extreme, neonatal medicine and cardiology have at least a 50% greater lifetime income than endocrinology or adolescent medicine. In other words, some subspecialists will earn approximately twice as much as other subspecialists during their lifetime. It is unknown what impact this income inequality among the subspecialties may have on the decisions of those who want to pursue fellowship training of some type.

The differences in lifetime income among the subspecialties may be of special interest to leaders in academic medicine as they search for strategies to increase applicants to many of the lower-paid fields.^23,24^ The higher-paid subspecialties are those with greater use of procedures, raising the possibility that the type of work conducted, not only remuneration, may be a significant factor underlying these variances in subspecialty desirability.

However, for many subspecialists, clinical care is only a fraction of their professional time commitments. Yet, it appears that the clinically based differences in compensation carry over to all other aspects of work, whether they be research, education, or service related. In other words, once a compensation range is determined for a specific subspecialty, all work is paid at the rate linked to the clinical care they provide. Differential pay for the same work in education, research, and administration has become the norm in our system. Academic leaders may want to explore the rationale for paying different subspecialists different rates for similar, nonclinical work or determining the potential for greater subsidization of the nonprocedural-based subspecialties.

### Limitations

This study has limitations. While there are data describing the mean educational debt among medical school students at the time of graduation,^25^ there is great variability among individuals and potentially among those choosing each subspecialty or engaging in private or academic employment. As such, debt cannot be accurately reflected or generalized for the purposes of this study without adding confounding to the results of each subspecialty. For our analysis, we have not included educational debt in our calculations of potential lifetime earnings so that no differential impact of the potentially nonrandom distribution of debt among general pediatrics or specific subspecialties will affect results. Further, the primary outcome of the study is income among pediatricians and does not include any other potential expenditures.

## Conclusions

The findings of this quality improvement study suggest that the difference in lifetime compensation between many pediatric subspecialties and general pediatrics is not as large as previously reported. A better understanding of the panoply of issues affecting the decisions of trainees to pursue subspecialty training in general and a specific subspecialty in particular is needed. Assumptions without data regarding the relative importance of some factors (ie, compensation) over others in this decision process may lead to ineffective programs and policy decisions intended to ameliorate perceived shortages. Realizing the full picture of compensation inequality and the role of other variables in work and lifestyle associated with the different pediatric subspecialties may help leaders to better appreciate the complexity of the issues at hand. Exploration of the rationale for payment of educational, research, and administrative effort based on clinical subspecialty might be a good place to start. Hopefully, this will also lead to evidence-based policy recommendations with higher likelihood of success.

## Notes

Supplement.
Data Sharing Statement
