# Supplementary material for: Comparison of Calculations of the Financial Impact of Fellowship Training by Data Source
Source: JAMA Netw Open. 2023 Jul 28;6(7):e2326639. doi: 10.1001/jamanetworkopen.2023.26639 (PMC10383003; doi:10.1001/jamanetworkopen.2023.26639)
Supplement: Supplement. — Data Sharing Statement [file jamanetwopen-e2326639-s001.pdf]

Data Sharing Statement

Freed. Comparison of Calculations of the Financial Impact of Fellowship Training by Data Source. *JAMA Netw Open*. Published July 28, 2023.
doi:10.1001/jamanetworkopen.2023.26639

Data
Data available: No
